# A cross-sectional survey of potential factors, motivations, and barriers influencing research participation and retention among people who use drugs in the rural USA

**DOI:** 10.1186/s13063-021-05919-w

**Published:** 2021-12-20

**Authors:** Angela T. Hetrick, April M. Young, Miriam R. Elman, Sarann Bielavitz, Rhonda L. Alexander, Morgan Brown, Elizabeth Needham Waddell, P. Todd Korthuis, Kathryn E. Lancaster

**Affiliations:** 1 Division of Epidemiology, College of Public Health, The Ohio State University, Columbus, USA; 2 Department of Epidemiology, University of Kentucky, Lexington, USA; 3 Center on Drug and Alcohol Research, University of Kentucky, Lexington, USA; 4 Oregon Health & Science University-Portland State University School of Public Health, Portland, USA; 5 Department of Medicine, Section of Addiction Medicine, Oregon Health & Science University, Portland, USA

**Keywords:** Rural, Substance use, Recruitment, Opioid, Injection drug use, Participant retention

## Abstract

**Background:**

Despite high morbidity and mortality among people who use drugs (PWUD) in rural America, most research is conducted within urban areas. Our objective was to describe influencing factors, motivations, and barriers to research participation and retention among rural PWUD.

**Methods:**

We recruited 255 eligible participants from community outreach and community-based, epidemiologic research cohorts from April to July 2019 to participate in a cross-sectional survey. Eligible participants reported opioid or injection drug use to get high within 30 days and resided in high-needs rural counties in Oregon, Kentucky, and Ohio. We aggregated response rankings to identify salient influences, motivations, and barriers. We estimated prevalence ratios to assess for gender, preferred drug use, and geographic differences using log-binomial models.

**Results:**

Most participants were male (55%) and preferred methamphetamine (36%) over heroin (35%). Participants reported confidentiality, amount of financial compensation, and time required as primary influential factors for research participation. Primary motivations for participation include financial compensation, free HIV/HCV testing, and contribution to research. Changed or false participant contact information and transportation are principal barriers to retention. Respondents who prefer methamphetamines over heroin reported being influenced by the purpose and use of their information (PR = 1.12; 95% CI: 1.00, 1.26). Females and Oregonians (versus Appalachians) reported knowing and wanting to help the research team as participation motivation (PR = 1.57; 95% CI: 1.09, 2.26 and PR = 2.12; 95% CI: 1.51, 2.99).

**Conclusions:**

Beyond financial compensation, researchers should emphasize confidentiality, offer testing and linkage with care, use several contact methods, aid transportation, and accommodate demographic differences to improve research participation and retention among rural PWUD.

**Supplementary Information:**

The online version contains supplementary material available at 10.1186/s13063-021-05919-w.

## Introduction

The rural United States (U.S.) is in the midst of an ongoing substance use disorder epidemic. In 2019, one in five Americans used an illicit drug and 70,630 drug overdose deaths occurred in the U.S, of which 71% were opioid-related [1, 2]. Further exacerbating the substance use epidemic is the prediction of the opioid epidemic’s “4th wave” or the rise in methamphetamine use among people with opioid use disorders [3–6]. People who use drugs (PWUD) such as opioids and methamphetamines face rising rates of HCV, HIV, and other chronic health conditions [7–9]. When rural PWUD attempt to access treatment for these health conditions, they face transportation barriers, stigma from healthcare providers, and a shortage of providers who offer care for substance use disorders (SUD) and their associated harms (e.g., HCV and HIV) [8, 10–12].

Despite high morbidity and mortality among PWUD in rural America, most clinical research is conducted among urban residents [13]. Results from clinical studies may not always translate to rural communities because of the various demographic, sociocultural, and infrastructural differences [14–17]. PWUD in rural communities may benefit from participation in research through otherwise unavailable access to SUD knowledge, specialists, and medical facilities, the receipt of new and effective treatments and medical care, and supported linkage to community SUD resources and programs [18, 19]. Although research studies such as clinical trials may provide valuable care and treatment to rural PWUD, retention and recruitment remains challenging [15, 20, 21]. Those in rural communities note mistrust and fear as contributors to low recruitment, and rural residents have a lower likelihood of awareness of opportunities to participate in research compared to their urban counterparts [21, 22].

Factors affecting clinical trial recruitment and retention can be categorized using the Ickovics and Mieslers multifactorial framework: the individual, treatment regimen, patient-provider relationship, clinical setting, and the disease [23]. There is mixed evidence for the presence of individual level differences in research enrollment and retention between genders. The National Institute on Drug Abuse (NIDA) Treatment Clinical Trials Network (CTN) did not identify proportional differences in enrollment and retention between genders, and willingness to participate in an HCV vaccine trial among PWUD did not differ by gender [24–26]. However, other studies suggest that women are underrepresented in HCV and HIV clinical research [27], and gender differences in utilization of harm reduction services exist among rural PWUD [28]. Further, there is some evidence to indicate that considerations for participating in research studies differ by gender [29, 30]. An individual’s drug preference for methamphetamine over heroin may also impact trust in research due to adverse effects such as elevated paranoia and suspiciousness associated with methamphetamine use [31]. Patient-provider relationship factors such as stigmatizing attitudes unique to certain drugs [32–34] could result in variations in the perceived judgment by staff as a factor affecting participation in research studies, including studies irrelevant to substance use.

Our study’s primary objective is to describe the influencing factors, motivations, and barriers of rural PWUD in participation and retention in research studies. The study’s secondary objective is to examine variation in influencing factors, motivations, and barriers across geographic regions, gender, and substance use to inform retention and recruitment strategies.

## Methods

### Study setting

We conducted a cross-sectional survey from April to July 2019 in rural areas of Kentucky, Ohio, and Oregon, where each state has an established research infrastructure through the National Rural Opioid Initiative [35]. Rural study sites in Eastern Kentucky and Southeastern Ohio are located in Appalachia, a cultural and geographical region that spans 13 states from New York to Mississippi [36]. The Oregon rural study sites in the Pacific Northwest include both coastal and interior communities in large, sparsely populated counties. The populations in these areas of Appalachia and Oregon are predominantly White, and an estimated 12–38% of the population lives below the poverty line, compared to the U.S. national average of 10.5% [37, 38]. These rural communities are at increased vulnerability for HIV and HCV transmission due to high injection drug use rates and inadequate healthcare infrastructures [39–41].

### Study design

The cross-sectional, multi-state survey was part of the formative phase of the Peer-based Retention of People who Use Drugs in Rural Research (PROUD-R^2^) study that tests rural peers’ ability to improve study retention (ClinicalTrials.gov identifier: NCT03885024) [42]. We estimated that a sample size of 225 participants, or approximately 75 participants per site, would provide a saturation of responses to inform the central phase of PROUD-R^2^.

Eligible participants were at least 18 years of age, injected any drug or used opioids through non-injection methods (such as smoking, inhaling, snorting, or swallowing) to get high within the past 30 days, and lived within rural counties associated with each study site. We recruited participants using convenience sampling at syringe service programs, local health departments, community-based settings, and through concurrent epidemiologic studies. We obtained informed consent from all participants and provided each participant with $20 cash or a gift card as reimbursement for survey participation. The survey was interviewer-administered in Kentucky and Oregon and self-administered in Ohio. In Ohio, trained study staff followed an IRB-approved script to recruit and verbally determine participant eligibility as persons departed a local, weekly syringe service program. In Kentucky, individuals who had participated in a previous study on drug use in the study region and who had consented to be contacted about future research opportunities were contacted by the study staff and invited to participate using an IRB-approved recruitment script. To those who were interested in participating, staff verbally administered each question from the eligibility screening survey and recorded their responses in the survey instrument. In some cases, individuals who were screened and/or enrolled informally told their peers about the study who then called to express interest in participating and were screened. In Oregon, potential participants were recruited from syringe service programs through flyers and staff and peer referrals. Survey instruments were administered and responses electronically recorded by research staff, as in Kentucky. We used self-administration survey methods in Ohio to ensure confidentiality of participant responses as data collection occurred in a shared room of a local syringe service program. In Kentucky and Ohio, we collected data using Qualtrics software, Version June 2019 (Qualtrics, Provo, UT). In Oregon, study data were collected using REDCap electronic data capture tools hosted at Oregon Health & Science University [Grant#: UL1TR002369 ][43].

### Data collection

We constructed our survey based on prior literature on clinical trial participation among PWUD and adapted from previous assessments of vaccine trial willingness [24, 44, 45]. Questions were adapted to ask without reference to a specific vaccine or trial product to more broadly understand factors, motivations, and barriers to research participation and retention among rural PWUD. The Community Advisory Board of the Kentucky CARE2HOPE study and peer recovery support specialists of the Oregon OR-HOPE study reviewed and approved the survey’s final version to confirm the appropriateness of the survey.

Participants provided the following demographic information: age, gender, education, race, and ethnicity. We assessed gender using the construct, “What is your gender?” in alignment with recommendations to use gender as opposed to sex when reporting psychosocial or cultural factors [46]. We also asked participants about recent drug use, “Have you ever injected drugs to get high?”, “Which drugs have you injected in the past 30 days to get high?”, and “Which is your drug of choice for getting high?”.

To capture each participant’s history with research studies, we asked, “Before today, have you ever participated in a research study?” Participants who selected “yes” were prompted to select all that apply to, “What did the research you participated in involve?” Response options included “in-person survey(s) or interview(s),” “telephone survey(s),” “testing for a disease or health condition (not including a urine drug test),” “a clinical trial testing a new drug, treatment or device,” “follow-up appointments for surveys and/or testing,” “financial incentive (i.e., money or gift card given for participation),” and “other (please specify).”

We collected data on (1) influencing factors for research participation, (2) motivations for participating in research, and (3) barriers to attend follow-up research appointments through a series of nominal response option questions in which the participant could “select all that apply.” To elicit factors that influenced participants’ decision to participate in a research study, we asked, “What are some of the things that people who use drugs in this community may consider when deciding to participate in a research study?” [15 response options]. We inquired, “What are some of the reasons that people who use drugs in this community may decide to participate in a research study?” [10 response options] to obtain their motivations for participation in research. To assess barriers to retention in attending follow-up research appointments, we asked, “What do you think are some of the challenges to getting people to come back for follow-up appointments?” [11 response options]. Each question included an “other” response option that, if selected, prompted participants to specify a response not listed. Complete response options for all three questions are listed in Tables 3, 4, and 5. If a participant selected more than three responses to the above questions, they were prompted to “Select their top 3” options by specifying their “1st choice,” 2nd choice,” and “3rd choice” from their previously selected responses, hereafter referred to as primary, secondary, and tertiary responses. Their remaining selected responses were categorized as “unranked.” For participants that selected a single response, we categorized the response as “primary.” For participants that selected only two to three responses we categorized their responses as “top three, unranked.” If a participant ranked only one to two responses, the remaining responses were categorized as “unranked.”

### Statistical analyses

Participant sociodemographics and drug use characteristics were summarized using descriptive statistics. We excluded participants from analyses if they were missing a response for one or more of the eligibility questions. To visually represent and compare the ranking of participant responses, we used diverging stacked bar charts [47]. We displayed survey items with the highest to lowest frequency of primary, secondary, and tertiary responses. We also included counts of selected, but unranked, responses for each survey item.

We aggregated the survey responses into 36 binary dependent variables to analyze the differences of influencing factors, motivations for participation, and barriers to retention in research among subgroups of the study population. Due to small cell sizes, we condensed the rankings of each response into dichotomous variables (“selected,” “not selected”) that represented a participant’s response to selecting items as essential influencing factors, motivations, or barriers.

Of the demographic variables available, we selected and dichotomized three variables a priori with the potential to inform future research recruitment and retention protocols. Specifically, we conducted comparisons by gender given mixed findings from previous research on the association between gender and reasons for research participation [29, 30]. Analyses by geographic location and type of drug use address gaps in the literature on how these factors may impact research participation. The latter is especially important given the evolving drug epidemic in rural areas from one that is predominated by opioids to one of polysubstance use involving methamphetamine [48]. We assessed differences in the dependent variable by the independent variables of gender (male, female), region (Appalachia, Oregon), and preferred drug of choice (heroin, methamphetamine). We combined Kentucky and Ohio into a single “Appalachia” group for several conceptual and statistical reasons: the stratified prevalence ratios of Kentucky and Ohio were similar when compared to Oregon, the Kentucky and Ohio research sites are geographically close, and both are in the Appalachian region. Of the preferred choice of drug options included in our survey, we compared heroin and methamphetamine as most participants selected one or the other as their ideal drug of choice. To ensure that “preferred drug of choice” reflected actual use, we verified that most participants had access to their preferred drug of choice by generating cross tabulations with their preferred drug of choice and reported substance use in the past 30 days.

We selected log-binomial regression a priori to assess differences in site, gender, and preferred drug use for each of the 36 selected responses. Prevalence ratios (PR) and corresponding 95% confidence intervals (CI) were estimated for each bivariable model. To reduce bias and improve model precision, only survey items with at least ten responses at each level of the binary dependent variable were modeled [49–51].

We aggregated and analyzed data using SAS software version 9.4 (SAS Institute Inc., Cary, NC) at Oregon Health and Science University and the Ohio State University. Plots were developed with the “HH” package in R version 4.0.2 (R Core Team, Vienna, Austria )[52, 53]. The Ohio State University Institutional Review Board, University of Kentucky Institutional Review Board, and Oregon Health and Science University Institutional Review Board approved this study.

## Results

### Participant characteristics

A total of 290 participants completed the survey. In Oregon and Kentucky, a total of 218 participants were screened and completed the survey, and 34 participants were ineligible, for reasons including not meeting drug use eligibility criteria (*n* = 28), living outside of the study location (*n* = 4), or missing information for all eligibility criteria (*n* = 2). In Ohio, a total of 72 participants were screened and completed the survey. Participant eligibility was assessed verbally using a recruitment and eligibility script, and the number of ineligible participants and reasons for ineligibility were not recorded. Furthermore, one Ohio participant was excluded from the analyses due to missing age and could not be confirmed to meet study eligibility. The final analytic sample contained 255 participants and included a complete set of responses for all independent and dependent variables.

The characteristics of the 255 participants included in our study are shown in Table 1. Most participants were from Kentucky (*n* = 105), then Oregon (*n* = 79), followed by Ohio (*n* = 71), and the median participant age was 37 years (IQR: (30, 45); range: (19, 72)). Most participants identified as male (55%), white (88%), and had at least a high school diploma/General Educational Development (GED) (73%). One participant identified their gender as “Transgender” and another participant identified as “Unknown/Unsure.” We excluded these participants from the analysis between gender differences to avoid applying unreliable findings to these subpopulations resulting from their small sample sizes. Further, if these two individuals were described in more detail demographically and behaviorally, they may be identifiable in their communities, and we wish to protect their confidentiality.

**Table 1:** Demographics of PWUD who completed a cross-sectional survey in rural Oregon, Ohio, and Kentucky, April–July 2019

	Total study population		Oregon		Kentucky		Ohio	
	(*n* = 255)		(*n* = 79)		(*n* = 105)		(*n* = 71)	
	*n*	*%*	*n*	*%*	*n*	*%*	*n*	*%*
Age								
Median (IQR)	37	*(30, 45)*	34	*(29, 43)*	37	*(30, 45)*	38	*(32, 45)*
(Range)		*(19, 72)*		*(19, 61)*		*(19, 72)*		*(25, 66)*
Gender								
Female	116	*45*	36	*46*	51	*49*	29	*41*
Male	137	*55*	43	*54*	54	*51*	40	*56*
Transgender	1	*0*	0	*0*	0	*0*	1	*1*
Unknown/unsure	1	*0*	0	*0*	0	*0*	1	*1*
Race								
White	225	*88*	60	*76*	104	*99*	61	*86*
Black/African American	5	*2*	2	*3*	0	*0*	3	*4*
American Indian/Alaskan Native	6	*2*	5	*6*	0	*0*	1	*1*
Mixed race	13	*5*	10	*13*	1	*1*	2	*3*
Other	6	*2*	2	*3*	0	*0*	4	*6*
Ethnicity								
Hispanic	14	*5*	11	*14*	1	*1*	2	*3*
Non-Hispanic	241	*95*	68	*86*	104	*99*	69	*97*
Education								
Less than high school	69	*27*	10	*13*	41	*39*	18	*25*
High school diploma or GED	106	*42*	36	*46*	40	*38*	30	*42*
Some college	59	*23*	29	*37*	19	*18*	11	*16*
Associate’s degree, trade, or technical college	16	*6*	3	*4*	4	*4*	9	*13*
Bachelor’s degree or higher	3	*1*	1	*1*	1	*1*	1	*1*
Missing	0	*0*	0	*0*	0	*0*	2	*3*
Highest level of education								
High school diploma or GED	184	*73*	69	*87*	64	*61*	51	*74*
Below high school diploma or GED	69	*27*	10	*13*	41	*39*	18	*25*

Nearly all participants had a lifetime history of injecting drugs to get high (93%) (Table 2). Most participants preferred either heroin or methamphetamine; both groups reported recent use (92% for heroin, 95% for methamphetamine), and both were the most commonly injected drugs in the past 30 days (57% and 61%, respectively). Other recently injected drugs included fentanyl (18%), buprenorphine (18%), painkillers (15%), cocaine/crack (8%), methadone (3%), and prescription anxiety drugs (3%).

**Table 2:** Drug use history among rural PWUD in Kentucky, Ohio, and Oregon, April 2019–July 2019

		Total study population (*n* = 255)		Oregon (*n* = 79)		Kentucky (*n* = 105)		Ohio (*n* = 71)	
		*n*	*%*	*n*	*%*	*n*	*%*	*n*	*%*
Ever injected drugs to get high									
Yes		237	*93*	75	*95*	95	*90*	67	*94*
No		18	*7*	4	*5*	10	*10*	4	*6*
Drugs injected to get high in past 30 days									
Methamphetamines		155	*61*	61	*77*	70	*67*	24	*34*
Heroin		144	*57*	42	*53*	48	*46*	54	*76*
Buprenorphine		47	*18*	5	*6*	33	*31*	9	*13*
Fentanyl		47	*18*	14	*18*	10	*10*	23	*32*
Painkillers		38	*15*	21	*27*	12	*11*	5	*7*
Cocaine/crack		21	*8*	11	*14*	6	*6*	4	*6*
Other^a^		11	*4*	10	*13*	0	*0*	1	*1*
Methadone		8	*3*	5	*6*	2	*2*	1	*1*
Prescription anxiety drugs		8	*3*	6	*8*	1	*1*	1	*1*
Gabapentin		1	*1*	1	*1*	0	*0*	0	*0*
Kratom^b^		1	*1*	1	*1*	0	*0*	-	*-*
Clonidine		0	*0*	0	*0*	0	*0*	0	*0*
Synthetics		0	*0*	0	*0*	0	*0*	0	*0*
Drug of choice to get high									
Methamphetamines		91	*36*	37	*47*	45	*43*	9	*13*
Heroin		90	*35*	33	*42*	24	*23*	33	*47*
Buprenorphine		14	*6*	0	*0*	12	*11*	2	*3*
Painkillers		13	*5*	2	*3*	11	*11*	0	*0*
Fentanyl		5	*2*	0	*0*	0	*0*	5	*7*
Other^c^		3	*1*	3	*4*	0	*0*	0	*0*
Gabapentin		2	*1*	0	*0*	0	*0*	2	*3*
Methadone		1	*1*	0	*0*	1	*1*	0	*0*
Cocaine/crack		1	*1*	0	*0*	1	*1*	0	*0*

^a^“Other” responses included “cannabis” (*n* = 9), “pot, lsd, mushrooms, acid” (*n* = 1) and one response was missing (*n* = 1)

^b^Kratom was not listed as a response option in the Ohio survey

^c^“Other” responses included “pot” (*n* = 2) and “lsd, doc, mushrooms” (*n* = 1)

Most participants had previously participated in a research study (64%). The most commonly reported types of research were “in-person survey or interview” (*n* = 152), research that included a financial incentive (*n* = 120), research testing for a disease or health condition (*n* = 110), and research with follow-up appointments for a survey/testing (*n* = 92). Few participants noted research experience with telephone surveys (*n* = 6), clinical trials (*n* = 5), and other (*n* = 6). Participants who selected “other” reported participating in a research study that included, “drug swa(b) test,” “filling out papers,” “Narcan,” “computer surveys,” “internet surveys,” and one participant left their response blank.

### Patterns of influencing factors in decision to participate in research studies

**Fig. 1:**
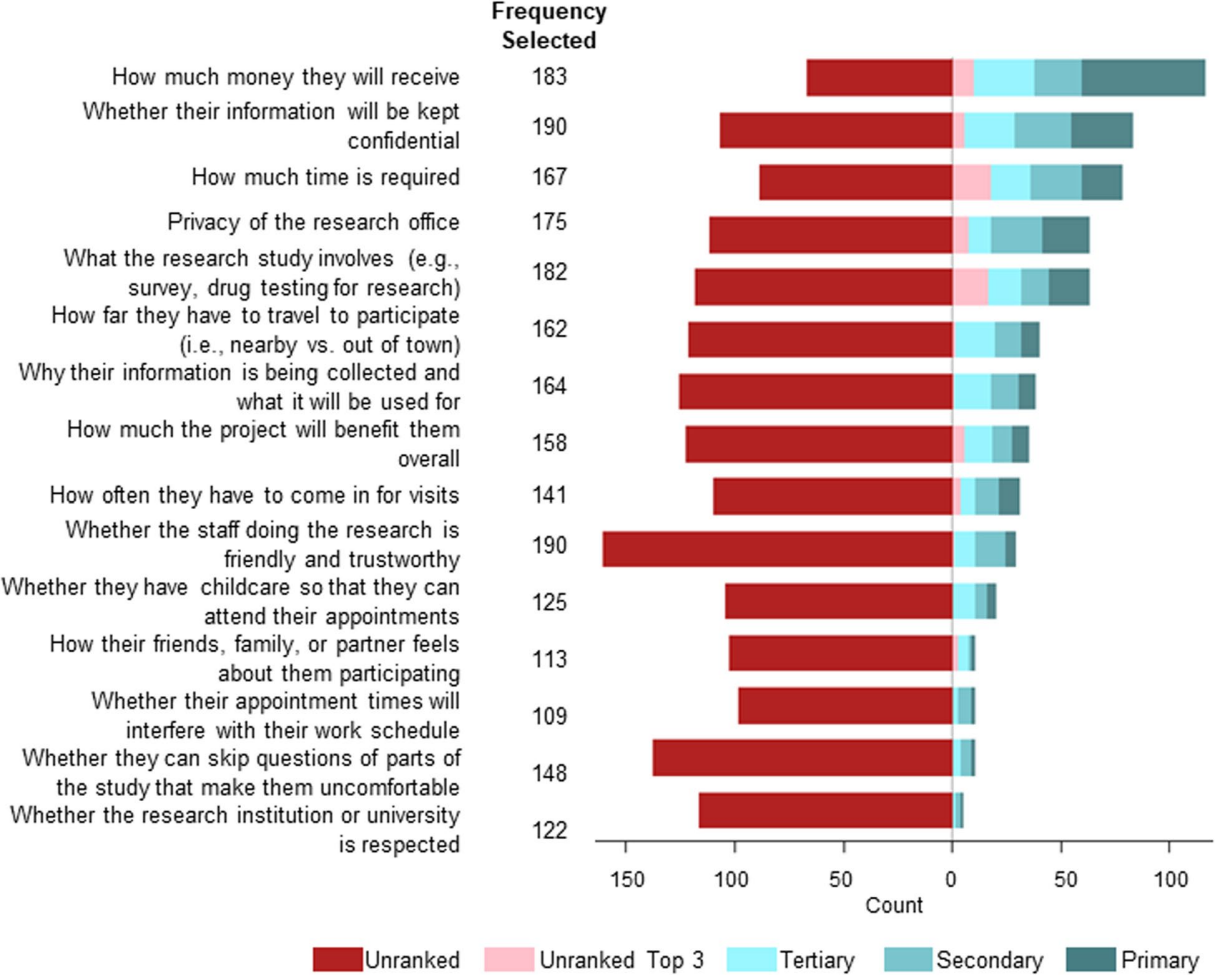
Ranked influencing factors for participating in research among PWUD in rural communities, April 2019–July 2019

The primary influencing factor for research participation was the amount of financial compensation received in exchange for participation (Fig. 1), followed by confidentiality of information. Other essential influencing factors among rural PWUD were the time required to participate in the research study and privacy of the research office. The influencing factor with the least number of ranked responses was whether the research institution or university was well-respected. Five participants selected “other” as an influencing factor for research participation. “Other” responses included “how much [the study] would help the community,” “whose approaching them and how they are approached,” and two responses were left blank.

In considering participation in research, Oregon respondents had a higher prevalence of selecting all influencing factors listed compared to Appalachian respondents. When compared to Appalachian respondents, Oregon respondents were more influenced by how much time is required for participation (PR = 1.42; 95% CI: 1.21, 1.67) and the frequency of research appointments (Oregon prevalence: 87%, Appalachian prevalence: 41%, PR = 2.14; 95% CI: 1.75, 2.60). Oregon respondents also had a higher prevalence of noting schedule conflicts, such as whether they have childcare available to attend research appointments (Oregon prevalence: 65%, Appalachian prevalence: 42%, PR = 1.54; 95% CI: 1.21, 1.95), and if their appointment times will interfere with their work schedule (PR = 1.63; 95% CI: 1.24, 2.14). Privacy of the research office (PR = 1.32; 95% CI: 1.13, 1.53) and knowledge of why their information is being collected and what it will be used for (PR = 1.46; 95% CI: 1.24, 1.73) were increased concerns among Oregon compared to Appalachian respondents (Table 3).

**Table 3:** Influencing factors and motivators to research participation and barriers to retention: Oregon versus Appalachia PWUD

Survey item	Oregon (***n*** = 79)		Appalachia (***n*** = 176)		Prevalence ratio^**a,b**^	95% CI
	Selected	Not selected	Selected	Not selected		
*Influencing factors*						
What the research study involves (e.g., survey, drug testing for research)^c^	71	8	111	65	–	–
How much time is required	65	14	102	74	1.42*	(1.21, 1.67)
How often they have to come in for visits	69	10	72	104	2.14*	(1.75, 2.60)
How far they have to travel to participate (i.e., nearby vs. out of town)^c^	70	9	92	84	–	–
Privacy of the research office	65	14	110	66	1.32*	(1.13, 1.53)
Why their information is being collected and what it will be used for	65	14	99	77	1.46*	(1.24, 1.73)
Whether their information will be kept confidential^c^	72	7	118	58	–	–
Whether the staff doing the research is friendly and trustworthy^c^	76	3	114	62	–	–
Whether the research institution or university is respected	44	35	78	98	1.26	(0.97, 1.63)
Whether they can skip questions of parts of the study that make them uncomfortable	60	19	88	88	1.52*	(1.25, 1.84)
How much money they will receive^c^	70	9	113	63	–	–
How much the project will benefit them overall^c^	70	9	88	88	–	–
Whether their appointment times will interfere with their work schedule	46	33	63	113	1.63*	(1.24, 2.14)
Whether they have childcare so that they can attend their appointments	51	28	74	102	1.54*	(1.21, 1.95)
How their friends, family, or partner feels about them participating	49	30	64	112	1.71*	(1.31, 2.21)
*Motivations*						
Financial incentive (i.e., money or gift card given for participation^c^	79	0	134	42	–	–
They believe in the mission of the research and want to contribute	61	18	96	80	1.42*	(1.18, 1.70)
Their friends, family, or partner participates	65	14	97	79	1.49*	(1.26, 1.77)
They want to tell their story	53	26	86	90	1.37*	(1.11, 1.70)
They know someone on the research team and want to help them out	40	39	42	134	2.12*	(1.51, 2.99)
They want to learn about the topic	51	28	79	97	1.44*	(1.14, 1.81)
They would want to get free testing (for example, rapid tests for HIV & Hepatitis C) if it was offered as part of the study^c^	71	8	102	74	–	–
They would want to be linked with resources and/or follow-up testing if it was offered as part of the study	69	10	103	73	1.49*	(1.28, 1.73)
They would want to try a new treatment if it was offered as part of the study	68	11	105	71	1.44*	(1.24, 1.68)
Their friends, family, or partner pressures them to participate so that they can share the financial incentive	42	37	43	133	2.18*	(1.56, 3.03)
*Barriers*						
Not being able to get in touch with participants because their contact information changed^c^	74	5	133	43	–	–
Not being able to get in touch with participants because they gave false contact information when they started the study	46	33	100	76	1.02	(0.82, 1.29)
They may have trouble getting transportation for their appointments	64	15	124	52	1.15	(0.99, 1.33)
They may have trouble being able to show up at a specific appointment time^c^	72	7	108	68	–	–
They may have trouble getting to their appointment because of their work schedule	51	28	71	105	1.60*	(1.26, 2.04)
They may have trouble finding childcare so that they can go to their appointment	53	26	74	102	1.60*	(1.26, 2.01)
They may have concerns about confidentiality and privacy	54	25	79	97	1.52*	(1.22, 1.90)
They may be afraid that the staff would judge them if they are still using drugs	41	38	76	100	1.20	(0.92, 1.58)
They may have stopped using drugs and no longer think the study is relevant to them	47	32	74	102	1.42*	(1.10, 1.82)
They are in a drug treatment or recovery facility and are unable to be contacted by research staff	55	24	92	84	1.33*	(1.09, 1.63)
Their friends, family, or partner may want them to stop participating	28	51	49	127	1.27	(0.87, 1.86)

^a^Kentucky and Ohio sites were combined to represent the referent group of “Appalachia”

^b^The level of response for each survey item was dichotomized into “selected” or “not selected” to generate prevalence ratios

^c^Oregon cell sizes for “not selected” were < 10; analysis not performed

* Significant at *α* =0.05 level

Compared to males (*n* = 137), female participants (*n* = 116) were more influenced to participate if they could skip uncomfortable questions of parts of the study (PR = 1.34; 95% CI: 1.09, 1.65) and if their information would be kept confidential (prevalence, females: 79%; males: 70%; PR = 1.15; 95% CI: 0.99, 1.32) (Table 4).

**Table 4:** Influencing factors and motivators to research participation and barriers to retention among PWUD: gender differences

Survey item	Male (***n*** = 137)		Female (***n*** = 116)		Prevalence ratio^**a,b**^	95% CI
	Selected	Not selected	Selected	Not selected		
*Influencing factors*						
What the research study involves (e.g., survey, drug testing for research)	100	37	81	35	0.98	(0.84, 1.15)
How much time is required	87	50	72	44	1.02	(0.85, 1.23)
How often they have to come in for visits	78	59	61	55	0.98	(0.78, 1.22)
How far they have to travel to participate (i.e., nearby vs. out of town)	85	52	76	40	1.09	(0.90, 1.31)
Privacy of the research office	92	45	81	35	1.18	(0.70, 2.01)
Why their information is being collected and what it will be used for	86	49	77	39	1.09	(0.91, 1.31)
Whether their information will be kept confidential	97	40	92	24	1.15	(0.99, 1.32)
Whether the staff doing the research is friendly and trustworthy	102	35	87	29	1.03	(0.89, 1.19)
Whether the research institution or university is respected	65	72	57	59	1.05	(0.81, 1.36)
Whether they can skip questions of parts of the study that make them uncomfortable	70	67	77	39	1.34*	(1.09, 1.65)
How much money they will receive	99	38	82	34	1.01	(0.87, 1.18)
How much the project will benefit them overall	81	56	77	39	1.14	(0.94, 1.38)
Whether their appointment times will interfere with their work schedule	64	73	45	71	0.84	(0.63, 1.13)
Whether they have childcare so that they can attend their appointments	66	71	59	57	1.07	(0.83, 1.38)
How their friends, family, or partner feels about them participating	68	69	45	71	0.79	(0.60, 1.05)
*Motivations*						
Financial incentive (i.e., money or gift card given for participation)	110	27	100	16	1.10	(0.99, 1.23)
They believe in the mission of the research and want to contribute	86	51	71	45	0.99	(0.81, 1.20)
Their friends, family, or partner participates	89	48	72	44	0.98	(0.82, 1.19)
They want to tell their story	77	60	62	54	0.96	(0.77, 1.21)
They know someone on the research team and want to help them out	36	101	45	71	1.57*	(1.09, 2.26)
They want to learn about the topic	73	64	56	60	0.94	(0.74, 1.20)
They would want to get free testing (for example, rapid tests for HIV & Hepatitis C) if it was offered as part of the study	93	44	79	37	1.03	(0.87, 1.22)
They would want to be linked with resources and/or follow-up testing if it was offered as part of the study	93	44	78	38	1.02	(0.86, 1.21)
They would want to try a new treatment if it was offered as part of the study	93	44	79	37	1.03	(0.87, 1.22)
Their friends, family, or partner pressures them to participate so that they can share the financial incentive	50	87	35	81	0.84	(0.59, 1.20)
*Barriers*						
Not being able to get in touch with participants because their contact information changed	111	26	94	22	1.03	(0.91, 1.16)
Not being able to get in touch with participants because they gave false contact information when they started the study	76	61	69	47	1.11	(0.90, 1.37)
They may have trouble getting transportation for their appointments	97	40	89	27	1.12	(0.96, 1.30)
They may have trouble being able to show up at a specific appointment time	94	43	85	31	1.09	(0.93, 1.28)
They may have trouble getting to their appointment because of their work schedule	66	71	55	61	1.03	(0.79, 1.33)
They may have trouble finding childcare so that they can go to their appointment	62	75	65	51	1.26	(0.98, 1.61)
They may have concerns about confidentiality and privacy	70	67	62	54	1.08	(0.86, 1.37)
They may be afraid that the staff would judge them if they are still using drugs	60	77	56	60	1.15	(0.88, 1.50)
They may have stopped using drugs and no longer think the study is relevant to them	63	74	57	59	1.11	(0.86, 1.44)
They are in a drug treatment or recovery facility and are unable to be contacted by research staff	81	56	65	51	0.98	(0.79, 1.21)
Their friends, family, or partner may want them to stop participating	38	99	39	77	1.23	(0.85, 1.79)

^a^Male is the referent group

^b^The level of response for each survey item was dichotomized into “selected” or “not selected” to generate prevalence ratios

*Significant at *α* = 0.05 level

Compared to those who selected heroin (*n* = 90) as their preferred drug of choice, respondents who selected methamphetamine (*n* = 91) were more influenced by the privacy of the research office (PR = 1.10; 95% CI: 0.99, 1.20), knowing why their information is collected and what it will be used for (prevalence, heroin: 59%, methamphetamine: 74%; PR = 1.12; 95% CI: 1.00, 1.26), and the confidentiality of their information (PR = 1.07; 95% CI: 0.99, 1.16) (Table 5).

**Table 5:** Influencing factors and motivators to research participation and barriers to retention: drug of choice differences, heroin vs. methamphetamine

Survey item	Heroin (***n*** = 90)		Methamphetamine (***n*** = 91)		Prevalence ratio^**a,b**^	95% CI
	Selected	not selected	selected	not selected		
*Influencing factors*						
What the research study involves (e.g., survey, drug testing for research)	69	21	68	23	0.96	(0.86, 1.06)
How much time is required	67	23	58	33	0.91	(0.79, 1.03)
How often they have to come in for visits	57	33	56	35	0.90	(0.78, 1.05)
How far they have to travel to participate (i.e., nearby vs. out of town)	58	32	67	24	1.01	(0.90, 1.13)
Privacy of the research office	58	32	73	18	1.10	(0.99, 1.20)
Why their information is being collected and what it will be used for	53	37	67	24	1.12*	(1.00, 1.26)
Whether their information will be kept confidential	63	27	80	11	1.07	(0.99, 1.16)
Whether the staff doing the research is friendly and trustworthy^c^	62	28	82	9	–	–
Whether the research institution or university is respected	39	51	51	40	1.25*	(1.08, 1.43)
Whether they can skip questions of parts of the study that make them uncomfortable	51	39	61	30	1.04	(0.91, 1.19)
How much money they will receive	64	26	75	16	1.01	(0.92, 1.11)
How much the project will benefit them overall	58	32	68	23	0.93	(0.82, 1.05)
Whether their appointment times will interfere with their work schedule	37	53	42	49	1.07	(0.88, 1.30)
Whether they have childcare so that they can attend their appointments	41	49	51	40	1.08	(0.91, 1.28)
How their friends, family, or partner feels about them participating	38	52	46	45	1.09	(0.90, 1.31)
*Motivations*						
Financial incentive (i.e., money or gift card given for participation	79	11	81	10	0.97	(0.91, 1.04)
They believe in the mission of the research and want to contribute	61	29	64	27	0.93	(0.82, 1.06)
Their friends, family, or partner participates	63	27	67	24	0.93	(0.82, 1.04)
They want to tell their story	49	41	54	37	1.03	(0.88, 1.20)
They know someone on the research team and want to help them out	27	63	37	54	1.00	(0.79, 1.28)
They want to learn about the topic	43	47	57	34	1.05	(0.90, 1.23)
They would want to get free testing (for example, rapid tests for HIV & Hepatitis C) if it was offered as part of the study	55	35	78	13	1.08	(0.99, 1.18)
They would want to be linked with resources and/or follow-up testing if it was offered as part of the study	55	35	73	18	1.09	(0.99, 1.21)
They would want to try a new treatment if it was offered as part of the study	59	31	76	15	1.03	(0.93, 1.13)
Their friends, family, or partner pressures them to participate so that they can share the financial incentive	34	56	32	59	0.88	(0.69, 1.13)
*Barriers*						
Not being able to get in touch with participants because their contact information changed	72	18	80	11	1.00	(0.93, 1.08)
Not being able to get in touch with participants because they gave false contact information when they started the study	51	39	61	30	0.99	(0.87, 1.14)
They may have trouble getting transportation for their appointments	68	22	71	20	0.99	(0.89, 1.09)
They may have trouble being able to show up at a specific appointment time	62	28	72	19	1.05	(0.95, 1.16)
They may have trouble getting to their appointment because of their work schedule	44	46	46	45	1.04	(0.89, 1.21)
They may have trouble finding childcare so that they can go to their appointment	47	43	49	42	0.94	(0.79, 1.12)
They may have concerns about confidentiality and privacy	43	47	60	31	1.09	(0.95, 1.26)
They may be afraid that the staff would judge them if they are still using drugs	38	52	48	43	1.09	(0.91, 1.31)
They may have stopped using drugs and no longer think the study is relevant to them	40	50	56	35	1.01	(0.86, 1.19)
They are in a drug treatment or recovery facility and are unable to be contacted by research staff	49	41	60	31	1.09	(0.96, 1.25)
Their friends, family, or partner may want them to stop participating	29	61	28	63	0.94	(0.71, 1.24)

^a^Heroin as the preferred drug of choice is the referent group

^b^The level of response for each survey item was dichotomized into “selected” or “not selected” to generate prevalence ratios

^c^Cell sizes for “not selected” were < 10; analysis not performed

* Significant at *α* = 0.05 level

### Patterns of motivations for research participation

**Fig. 2:**
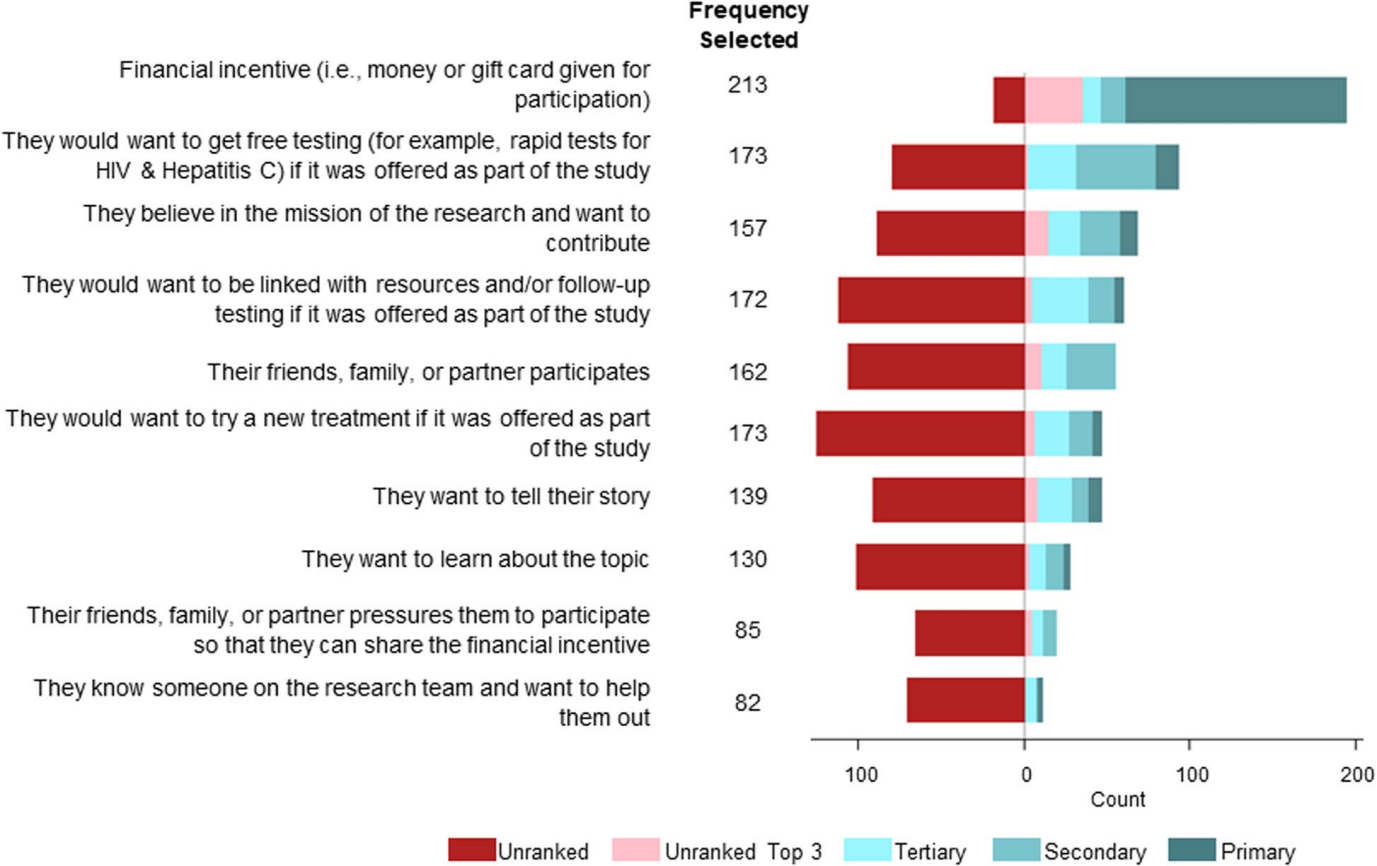
Ranked motivators for joining a research study among PWUD in rural communities, April 2019–July 2019

Financial compensation was the primary motivator for participation, followed by free diagnostic testing for infectious diseases such as HCV and HIV, and believing in the mission of the research and wanting to contribute (Fig. 2). “Knowing a person on the research team and wanting to help them out” was the least selected motivator for participation. Fifteen participants selected “other” as a motivator for research participation, while two participants left the response blank. “Other” responses included “it’s convenient,” “people get tired of watching their friends die,” “people might participate because of their families,” and “some people want to come and talk about getting off drugs and talk about resources.”

Compared to Appalachia respondents, Oregon respondents had a higher prevalence of motivation to enroll in research if they believe in the mission of the research and want to contribute (PR = 1.42; 95% CI: 1.18, 1.70) and to tell their story (PR = 1.37; 95% CI: 1.11, 1.70). The prevalence of noting feeling pressured to participate by peers to share the financial incentive (PR = 2.18; 95% CI: 1.56, 3.03) and knowing someone on the research team and wanting to help them out (PR = 2.12; 95% CI: 1.51, 2.99) was over two times greater among Oregon respondents compared to Appalachian respondents (Table 3).

Female participants were nearly twice as likely to report being motivated to participate in a research study if they knew someone on the research team (PR = 1.81; 95% CI: 1.09, 2.26) and were marginally more motivated to participate if a financial incentive was offered (prevalence, females: 41%, males: 32%; PR = 1.10; 95% CI: 0.99, 1.23) (Table 4).

Respondents whose preferred drug of choice was methamphetamine had a higher prevalence of being motivated to participate in research if they would receive free diagnostic testing (PR = 1.08; 95% CI: 0.99, 1.18) and linkage to resources and follow-up testing as part of the study (PR = 1.09; 95% CI: 0.99, 1.21), compared to respondents whose preferred drug of choice was heroin (Table 5).

### Patterns of anticipated barriers for retention in follow-up research appointments

**Fig. 3:**
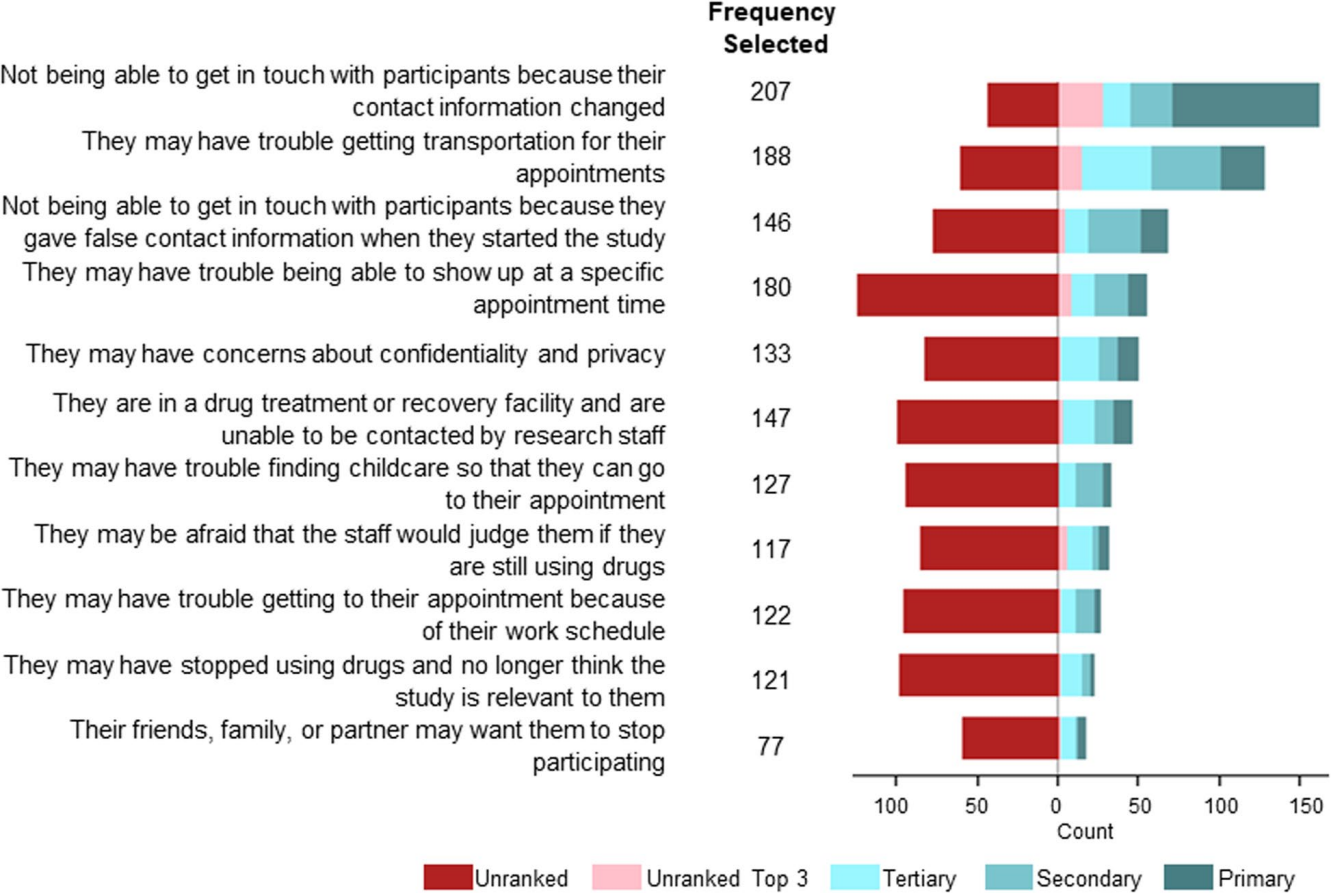
Ranked barriers to returning to follow-up appointments among PWUD in rural communities, April 2019–July 2019

Losing contact with participants due to changed contact information had the highest frequency of primary responses among barriers to returning to follow-up research appointments, followed by trouble obtaining transportation and sharing false contact information at their initial appointment (Fig. 3). The barrier with the lowest number of ranked responses among respondents was that their friends, family, or partner may want them to stop participating. Twenty participants selected “other” as a barrier for research participation, while three participants left the response blank. Their responses included, “being under the influence of drugs,” “if they did get clean this could be a trigger to come into the office,” “it’s not that important to them,” “they may be high and forget,” “people are scared to be tested for disease,” “people aren’t in stable environments and move a lot,” “people forget about the survey,” and “can’t force people to come in, they make their own choices.”

Reporting conflicts in returning to follow-up appointments due to work, finding childcare, and transportation were greatest among Oregon participants compared to Appalachian participants (PR = 1.60; 95% CI: 1.26, 2.04, PR = 1.60; 95% CI: 1.26, 2.01, and prevalence, Oregon: 81%, Appalachian: 70%; PR = 1.15; 95% CI: 0.99, 1.33, respectively). Oregon participants also had a higher prevalence of reporting privacy and confidentiality concerns (PR = 1.52; 95% CI: 1.22, 1.90) and becoming unreachable due to participation in a drug treatment program (PR = 1.33; 95% CI: 1.09, 1.63). Barriers that did not differ between Appalachian and Oregon participants included not being able to get in touch with participants because they provided false contact information and they may be afraid that the staff would judge them if they are still using drugs (Table 3).

Female participants were more likely to identify trouble finding transportation (PR = 1.12; 95% CI: 0.96, 1.30) and childcare (prevalence, females: 32%, males: 21%; PR = 1.26; 95% CI: 0.98, 1.61) as challenges to returning to follow-up research appointments (Table 4).

Participant-prioritized barriers did not differ by drug of choice (Table 5). Select rankings of factors, motivations, and barriers influencing research participation and retention varied by site (Additional File 2).

## Discussion

We identified several themes of rural PWUD considerations in deciding to participate and remain in research studies. The primary influencing factor and motivator for rural PWUD to participate in research is the amount and presence of financial compensation. Economic and social factors of the risk environment framework are determinants of substance use [54] and promote a disproportionate burden of substance use among people living below the federal poverty threshold [1]. A lack of assistance programs in areas where rural PWUD reside further exacerbate the economic needs of this population [55, 56]. The weight of financial compensation in our findings is consistent with studies of rural Kentucky PWUD populations and others that note receipt of financial compensation as positively associated with research participation and retention [24, 57, 58]. Lower economic status is also associated with poorer study retention; once enrolled in a longitudinal research study, PWUD who live below the federal poverty line are more likely to be lost to follow-up [59]. While financial compensation can be used to offset economic needs of participants and costs to participate such as travel and childcare, additional research is required to ascertain appropriate levels of financial compensation that do not coerce study participation among PWUD [60].

Financial need is an undercurrent relevant to other highly noted factors. Transportation was a major perceived barrier to retention among all participants irrespective of participant region, gender, or preferred drug use. Our findings align with previous studies that note transportation, or distance, as a primary barrier to retention among rural community members [20]. Western Oregon’s remote setting may increase transportation challenges. Oregon participants were more likely to consider factors and motivations for participation related to transportation concerns such as the frequency of research appointments and if their friend, family, or partner participates. Strategies to alleviate transportation challenges might include travel reimbursement, financial incentive amounts that account for transportation cost, or mobile or outreach models that bring the research to the participant.

Although financial incentive is the primary motivation for research participation among rural PWUD, our study supports other findings that motivations are multi-dimensional beyond monetary gains such as believing in the mission of the research and seeking linkage to care and other resources [57]. A lack of income paired with scarce medical care in rural locations may also explain the motivations noted by most rural PWUD to participate if linkage with resources and free testing are offered as part of the study. In the U.S., cost or lack of reimbursement by insurance companies and uncertainty about where to receive HIV care are reported as the primary barriers to HIV testing [61]. Due to a lack of healthcare assistance programs and primary care providers in rural communities, and stigma in healthcare settings, PWUD may prefer to access HCV and HIV screening offered by research studies [8, 55].

Privacy, confidentiality, and interaction quality with research staff are crucial influencing factors for PWUD in deciding to participate in research, likely due to stigma and the legal, employment, and inter-personal relationship consequences of substance use. Oregon respondents were more likely to be motivated to participate in research if they knew someone on the research staff. This finding may be related to the design of Oregon’s concurrent study (i.e., the Oregon HIV/HCV and Opioid Prevention and Engagement, or OR-HOPE) which employs peer recovery support specialists as study staff members [62]. While we found that most factors among rural PWUD did not differ between males and females, aligning with the findings of an analysis of 24 NIDA CTN trials [26], we found a notable difference in the importance of privacy between genders. Females reported an increased likelihood of indicating if their information would be kept confidential and whether they can skip questions that make them uncomfortable as important for participation. Female PWUD participants might be more concerned with privacy due to concerns of their reputation [30] or losing custody of their children if their drug use became publicly known [55] or due to anticipated distress around certain topics related to past trauma. Studies that recruit primarily rural, female PWUD should highlight confidentiality protections when obtaining informed consent and reiterate these protections throughout surveys, questionnaires, and other data collection items to encourage participation and improve comfort. In accordance with standards for best practice in research with vulnerable populations [63, 64], behavioral research studies can further support female rural PWUD by providing the opportunity to opt out of questions that make them uncomfortable.

Changing of participants’ contact information was a primary perceived barrier to returning to follow-up appointments and did not differ between participant region, gender, or preferred drug use. Our findings align with previous studies that note successfully contacting participants as a barrier to retention among rural community members and PWUD [65]. Obtaining information from participants about contact information of others (family, friends, etc.) who know how to reach them in case they cannot be contacted may improve retention [20]. The challenge of losing contact with participants may be alleviated by providing phone cards or other forms of contact reimbursement.

Participants who reported methamphetamine as their drug of choice to get high, as compared to those who selected heroin, had a higher prevalence of considering factors central to privacy and confidentiality. While not all of these factors met the threshold for statistical significance (*α* = 0.05), the positive measure of association speaks to a theme of distrust and privacy concerns present among those who prefer methamphetamine use. These findings align with a community-based study in Vancouver, Canada, that found those who use methamphetamine reported greater suspiciousness and paranoia compared to those who use opioids [31]. Methamphetamine-associated paranoia may magnify the general distrust of healthcare systems where PWUD frequently experience stigma [66, 67] and may exacerbate skepticism about the transparency of research which is already elevated among rural residents [68]. Research enrolling people who use methamphetamines in rural communities should tailor recruitment and retention strategies to emphasize confidentiality and privacy.

Our findings should be interpreted in light of several potential limitations. First, though our sample was drawn from U.S. rural communities in three states, findings may not be generalizable to rural communities outside of Appalachia and southwestern Oregon and may not be representative of all PWUD in the study communities due to our use of convenience sampling for data collection. Demographic characteristics of our sample align with those of previous studies of rural PWUD in the regions included in our study (i.e., mostly male, white, low education level, and age between 25 and 45 years [28, 69–72]); however, because county-level demographic information for PWUD in the study areas are not publicly available, we cannot fully assess the generalizability of our findings to rural PWUD in these locations. Second, the numerous response options provide crucial descriptive information on improving clinical trial recruitment and retention in rural areas but are likely correlated. Future work with large population-based samples will be needed for testing multiple hypotheses of multilevel factors. Still, our study found differences in factors for participation and retention between geographic locations and types of preferred drug use. The cross-sectional design of our study is a limitation in regard to capturing the challenges of enrollment and retention over time. We recommend that future longitudinal clinical research studies explore enrolled participants’ influencing factors and motivations for participation. Study staff should collect data on the reasons rural PWUD participants miss follow-up appointments among participants who are not lost to follow-up. Another limitation is the difference in administration methods between the three sites. The self-administered survey among Ohio respondents likely contributed to a lower selection of survey item responses. We believe the lower selection of responses biased the prevalence ratio comparisons between Oregon and Appalachia participants up and away from the null prevalence ratio value (i.e., 1.0). However, previous studies have demonstrated no difference in responses between interviewer- and self-administered surveys [73, 74]. Based on our results, further research is needed to determine if a difference in modes of survey administration is present among PWUD populations. A further limitation is that our results may differ by recency and duration of drug use; however, the need to reduce respondent burden precluded our ability to examine these variables of interest. Finally, participants were recruited in locations where epidemiological studies had already been recruiting rural PWUD; nearly two thirds of participants (64% overall, ranging from 42% in Ohio to 75% in Kentucky) reported previously participating in a research study. Therefore, our results may not adequately capture the perspectives of rural PWUD less familiar with or interested in research, which may differ. However, because our participants are more familiar with research, the reported factors may have been less hypothetical than with research naïve or adverse PWUD.

## Conclusions

Our findings contribute to the CTN’s focus on reaching underserved populations, such as rural PWUD, by identifying services such as testing, linkage to care, transportation, and factors such as privacy of clinic location and confidentiality of participant information that may enhance research participation and retention among this population. Research staff can address barriers to returning to follow-up appointments for rural PWUD by providing financial compensation, collecting detailed contact information from participants, and providing resources for transportation or by bringing the research to the participants through mobile or street outreach. Future longitudinal clinical research can leverage prominent influencing factors, motivations, and barriers to enhance participation and retention among rural PWUD.

### Supplementary Information

**Additional file 1:** PROUD-R2 Formative Survey.

**Additional file 2:** Potential factors, motivators, and barriers by study site.

## Data Availability

The data that support the findings of this study are not openly available due to the sensitive nature of the data and are available from the corresponding author upon reasonable request.

## Abbreviations

CTN: Clinical Trials Network
GED: General Education Development
HCV: Hepatitis C virus
NIDA: National Institute on Drug Abuse
PR: Prevalence ratio
PROUD-R^2^: Peer-based Retention of People who Use Drugs in Rural Research
PWUD: People who use drugs
SUD: Substance use disorder
U.S.: United States
