# Correction: A cross-sectional survey of potential factors, motivations, and barriers influencing research participation and retention among people who use drugs in the rural USA

**DOI:** 10.1186/s13063-023-07531-6

**Published:** 2023-09-28

**Authors:** Angela T. Hetrick, April M. Young, Miriam R. Elman, Sarann Bielavitz, Rhonda L. Alexander, Morgan Brown, Elizabeth Needham Waddell, P. Todd Korthuis, Kathryn E. Lancaster

**Affiliations:** 1 Division of Epidemiology, College of Public Health, The Ohio State University, Columbus, USA; 2 Department of Epidemiology, University of Kentucky, Lexington, USA; 3 Center On Drug and Alcohol Research, University of Kentucky, Lexington, USA; 4 Oregon Health & Science University-Portland State University School of Public Health, Portland, USA; 5 Department of Medicine, Section of Addiction Medicine, Oregon Health & Science University, Portland, USA

**Correction: BMC Trials 22, 948 (2021)**

**https://doi.org/10.1186/s13063-021-05919-w**

Following publication of the original article [1], we have been informed that a supplementary file was missing. This is now referenced at the end of the Results section (“Select rankings of factors, motivations, and barriers influencing research participation and retention varied by site (Additional File 2)”.

The original article has been corrected.
