# Supplementary material for: A cross-sectional survey of potential factors, motivations, and barriers influencing research participation and retention among people who use drugs in the rural USA
Source: Trials. 2021 Dec 20;22:948. doi: 10.1186/s13063-021-05919-w (PMC8690874; doi:10.1186/s13063-021-05919-w)
Supplement: Supplementary file 1 — Additional file 1: PROUD-R2 Formative Survey. [file 13063_2021_5919_MOESM1_ESM.docx]

PROUD-R^2^ Formative Survey

Q1.1 Interviewers Only: Interviewer – please enter the following details before beginning the survey with the participant.

SUBJECT Subject ID

________________________________________________________________

SUBJECT2 Subject ID (re-enter)

________________________________________________________________

SCRDATE Interview Date

________________________________________________________________

INT_ID Interviewer's Initials

________________________________________________________________

Q2.1 Thank you for your interest in the project. Today we will ask you some questions about how you and other people in the community feel about research and reasons why people may or may not participate in research studies. We appreciate your honesty and will keep your responses private. You can skip any questions that make you feel uncomfortable.  Your answers will help us better design research studies for people who live in rural communities like this one.

Q3.1
 First, we will start with some basic questions about your demographic characteristics.

How old are you?

________________________________________________________________

What is your gender?

o Male (1)

o Female (2)

o Transgender (3)

o Other (4) ________________________________________________

o Don't Know or Unsure (7)

How much school did you finish?

o Less than high school (1)

o High school diploma or GED (2)

o Some college (3)

o Associate degree, trade or technical school (4)

o Bachelor's degree, other 4 year college degree or more (5)

What race are you?

o White (1)

o African American or Black (2)

o American Indian (3)

o Alaskan Native (4)

o Asian or Pacific Islander or Native Hawaiian (5)

o African (6)

o Mixed Race (7)

o Other (8) ________________________________________________

Are you Hispanic or Latino?

o Yes (1)

o No (0)

o Don't Know (7)

What is the zip code where you have slept most in the past 30 days?

________________________________________________________________

In what city or town have you slept most in the past 30 days?

________________________________________________________________

In what county have you slept the most in the past 30 days?

________________________________________________________________

Q4.1 Thank you. Now, we will ask you some questions about your recent drug use.  Remember, this study is protected by a Certificate of Confidentiality from the Federal Government.  The Certificate protects your personal information from subpoena and other violations of your privacy.

Have you drank alcohol in the past 30 days?

o No (0)

o Yes (1)

o Don't Know (7)

Have you used tobacco in the past 30 days?

o No (0)

o Yes (1)

o Don't Know (7)

Have you ever used any drug to get high?

o No (0)

o Yes (1)

Which of the following drugs have you ever used to get high? (Check all that apply)

▢ Heroin (1)

▢ Street fentanyl or carfentanil powder (2)

▢ Opiate painkillers - like oxycodone, Percocet, Percodan, OxyContin, hydrocodone, Vicodin, Lorcet, Lortab, Norco, Morphine, Ultram, Dilaudid, Opana, T3s, fentanyl patch, etc. (3)

▢ Synthetics like U47700, U4, or "Pink" (4)

▢ Buprenorphine - like Suboxone, etc. (5)

▢ Methadone (6)

▢ Prescription anxiety drugs - like Xanax, Valium, Klonopin, etc. (7)

▢ Cocaine or crack (8)

▢ Methamphetamine, crystal meth (ice), or amphetamine (9)

▢ Gabapentin - like Neurontin (10)

▢ Clonidine (11)

▢ Kratom (12)

▢ Other (13) ________________________________________________

When did you last use heroin, fentanyl, carfentanil powder, opiate painkillers, or synthetics to get high? Enter as mm-dd-yyyy.

 It's ok if you don't remember the exact date, your best guess is fine.

________________________________________________________________

When did you last use buprenorphine -- like Suboxone, Subutex, etc. -- to get high? Enter as mm-dd-yyyy.

 It's ok if you don't remember the exact date, your best guess is fine.

________________________________________________________________

When did you last use methadone to get high? Enter as mm-dd-yyyy.

 It's ok if you don't remember the exact date, your best guess is fine.

________________________________________________________________

When did you last use methamphetamine, crystal meth, amphetamine, cocaine or crack to get high? Enter as mm-dd-yyyy.

It's ok if you don't remember the exact date, your best guess is fine

________________________________________________________________

Have you ever injected drugs to get high?

o No (0)

o Yes (1)

When did you last inject drugs to get high? Enter as mm-dd-yyyy.

 It's ok if you don't remember the exact date, your best guess is fine.

________________________________________________________________

Carry Forward Selected Choices - Entered Text from "Which of the following drugs have you ever used to get high? (Check all that apply)"

Which of the following drugs have you injected to get high in the past 30 days? (Check all that apply)

▢ Heroin (1)

▢ Street fentanyl or carfentanil powder (2)

▢ Opiate painkillers - like oxycodone, Percocet, Percodan, OxyContin, hydrocodone, Vicodin, Lorcet, Lortab, Norco, Morphine, Ultram, Dilaudid, Opana, T3s, fentanyl patch, etc. (3)

▢ Synthetics like U47700, U4, or "Pink" (4)

▢ Buprenorphine - like Suboxone, etc. (5)

▢ Methadone (6)

▢ Prescription anxiety drugs - like Xanax, Valium, Klonopin, etc. (7)

▢ Cocaine or crack (8)

▢ Methamphetamine, crystal meth (ice), or amphetamine (9)

▢ Gabapentin - like Neurontin (10)

▢ Clonidine (11)

▢ Kratom (12)

▢ Other (13)

Carry Forward Selected Choices - Entered Text from "Which of the following drugs have you ever used to get high? (Check all that apply)"

Currently, which is your drug of choice for getting high?
 Please choose one.  (Choose one)

o Heroin (1)

o Street fentanyl or carfentanil powder (2)

o Opiate painkillers - like oxycodone, Percocet, Percodan, OxyContin, hydrocodone, Vicodin, Lorcet, Lortab, Norco, Morphine, Ultram, Dilaudid, Opana, T3s, fentanyl patch, etc. (3)

o Synthetics like U47700, U4, or "Pink" (4)

o Buprenorphine - like Suboxone, etc. (5)

o Methadone (6)

o Prescription anxiety drugs - like Xanax, Valium, Klonopin, etc. (7)

o Cocaine or crack (8)

o Methamphetamine, crystal meth (ice), or amphetamine (9)

o Gabapentin - like Neurontin (10)

o Clonidine (11)

o Kratom (12)

o Other (13)

Q5.1 Thank you for completing the screener.  The staff person will now review your answers to determine if you are eligible.

How was this screening survey completed?

o In the study office (0)

o By phone call (1)

o By text messaging (2)

o At a cookout or study event (3)

o Don't Know (7)

Participant reports being ${SCRAGE/ChoiceTextEntryValue} years old.  Is this equal to or older than age 18?

o No (0)

o Yes (1)

Participant reports having slept most often in the past 30 days in:
Zipcode: ${SCRZIP/ChoiceTextEntryValue}

City/town: ${SCRTWN/ChoiceTextEntryValue}
County: ${SCRCNTY/ChoiceTextEntryValue}


Is this in the study area?

o No (0)

o Yes (1)

The participant last used opioids on ${SCROPDT/ChoiceTextEntryValue}.  Was this in the last 30 days?

o No (0)

o Yes (1)

The participant last used buprenorphine on ${SCRBUP/ChoiceTextEntryValue}.  Was this in the last 30 days?

o No (0)

o Yes (1)

The participant last used methadone on ${SCRMTD/ChoiceTextEntryValue}.  Was this in the last 30 days?

o No (0)

o Yes (1)

The participant last used stimulants on ${SCRSTDT/ChoiceTextEntryValue}.  Was this in the last 30 days?

o No (0)

o Yes (1)

The participant last injected drugs on ${SCRINDT/ChoiceTextEntryValue}.  Was this in the last 30 days?

o No (0)

o Yes (1)

Is the participant NOT eligible for any of the following reasons? (Check all that apply)

▢ Not currently enrolling people from their town/county (1)

▢ They have already participated (i.e., duplicate) (2)

▢ They seem to be coerced into participating by a peer (3)

▢ They seem to be falsifying information (4)

▢ They do not have the capacity to consent right now (5)

▢ They are being combative, threatening, or violent (6)

▢ Other (7) ________________________________________________

Now, we will switch gears and ask you some questions about how people who use drugs in this community feel about research and why they may or may not participate.

Before today, have you ever participated in a research study?

o No (0)

o Yes (1)

o Refuse to Answer (8)

Skip To: PARRES If Before today, have you ever participated in a research study? != Yes

PARTYP What did the research you participated in involve? (Check all that apply)

▢ In-person survey(s) or interview(s) (1)

▢ Telephone survey(s) (2)

▢ Testing for a disease or health condition (not including a urine drug test) (3)

▢ A clinical trial testing a new drug, treatment, or device (4)

▢ Follow-up appointments for surveys and/or testing (5)

▢ Financial incentive (i.e., money or gift card given for participation) (6)

▢ Other (7) ________________________________________________

Was substance use a focus of the study?

o No (0)

o Yes (1)

o Don't Know (7)

o Refuse to Answer (8)

What are some of the reasons that people who use drugs in this community may decide to participate in a research study? Select all that apply.

▢ Financial incentive (i.e., money or gift card given for participation) (1)

▢ They believe in the mission of the research and want to contribute (2)

▢ Their friends, family, or partner participates (3)

▢ They want to tell their story (4)

▢ They know someone on the research team and want to help them out (5)

▢ They want to learn about the topic (6)

▢ They would want to get free testing (for example, rapid tests for HIV & Hepatitis C) if it was offered as part of the study (7)

▢ They would want to be linked with resources and/or follow-up testing if it was offered as part of the study (8)

▢ They would want to try a new treatment if it was offered as part of the study (9)

▢ Their friends, family, or partner pressures them to participate so that they can share the financial incentive (i.e., money or gift card for participation) (10)

▢ Other (11) ________________________________________________

Skip To: PARCON If Selected Choices <= 3

Carry Forward Selected Choices from "What are some of the reasons that people who use drugs in this community may decide to participate in a research study? Select all that apply."

Select the top 3 reasons for why people who use drugs in this community may decide to participate in a research study.

| Top 3 Reasons: Decide to Participate in Research Study |
| --- |
| ______ Financial incentive (i.e., money or gift card given for participation) (x1) |
| ______ They believe in the mission of the research and want to contribute (x2) |
| ______ Their friends, family, or partner participates (x3) |
| ______ They want to tell their story (x4) |
| ______ They know someone on the research team and want to help them out (x5) |
| ______ They want to learn about the topic (x6) |
| ______ They would want to get free testing (for example, rapid tests for HIV & Hepatitis C) if it was offered as part of the study (x7) |
| ______ They would want to be linked with resources and/or follow-up testing if it was offered as part of the study (x8) |
| ______ They would want to try a new treatment if it was offered as part of the study (x9) |
| ______ Their friends, family, or partner pressures them to participate so that they can share the financial incentive (i.e., money or gift card for participation) (x10) |
| ______ Other (x11) |

PARCON What are some of the things that people who use drugs in this community may consider when deciding to participate in a research study? Select all that apply.

What the research study involves (e.g., survey, drug testing for research) (1)

▢ How much time is required (2)

▢ How often they have to come in for visits (3)

▢ How far they have to travel to participate (i.e., nearby vs. out of town) (4)

▢ Privacy of the research office (5)

▢ Why their information is being collected and what it will be used for (6)

▢ Whether their information will be kept confidential (7)

▢ Whether the staff doing the research is friendly and trustworthy (8)

▢ Whether the research institution or university is respected (9)

▢ Whether they can skip questions or parts of the study that make them uncomfortable (10)

▢ How much money they will receive (11)

▢ How much the project will benefit them overall (12)

▢ Whether their appointment times will interfere with their work schedule (13)

▢ Whether they have childcare so that they can attend their appointments (14)

▢ How their friends, family, or partner feels about them participating (15)

Other (16) ________________________________________________

Skip To: End of Block If Selected Choices <= 3

Carry Forward Selected Choices from "What are some of the things that people who use drugs in this community may consider when deciding to participate in a research study? Select all that apply."

Select the top 3 things that people who use drugs in this community may consider when deciding to participate in a research study.

| Top 3 Factors: Deciding to Participate in Research Study |
| --- |
| ______ What the research study involves (e.g., survey, drug testing for research) (x1) |
| ______ How much time is required (x2) |
| ______ How often they have to come in for visits (x3) |
| ______ How far they have to travel to participate (i.e., nearby vs. out of town) (x4) |
| ______ Privacy of the research office (x5) |
| ______ Why their information is being collected and what it will be used for (x6) |
| ______ Whether their information will be kept confidential (x7) |
| ______ Whether the staff doing the research is friendly and trustworthy (x8) |
| ______ Whether the research institution or university is respected (x9) |
| ______ Whether they can skip questions or parts of the study that make them uncomfortable (x10) |
| ______ How much money they will receive (x11) |
| ______ How much the project will benefit them overall (x12) |
| ______ Whether their appointment times will interfere with their work schedule (x13) |
| ______ Whether they have childcare so that they can attend their appointments (x14) |
| ______ How their friends, family, or partner feels about them participating (x15) |
| ______ Other (x16) |

Q9.1 Some research studies require participants to return for follow-up appointments. For example, in a study we are planning, we would like for people to start with a survey and then come back every 6 months for a follow-up survey so that we can learn how their behaviors and circumstances have changed over time.

What do you think are some of the challenges to getting people to come back for follow-up appointments? Select all that apply.

▢ Not being able to get in touch with participants because their contact information changed (1)

▢ Not being able to get in touch with participants because they gave false contact information when they started the study (2)

▢ They may have trouble getting transportation for their appointments (3)

▢ They may have trouble being able to show up at a specific appointment time (4)

▢ They may have trouble getting to their appointment because of their work schedule (5)

▢ They may have trouble finding childcare so that they can go to their appointment (6)

▢ They may have concerns about confidentiality and privacy (7)

▢ They may be afraid that the staff would judge them if they are still using drugs (8)

▢ They may have stopped using drugs and no longer think the study is relevant to them (9)

▢ They are in a drug treatment or recovery facility and are unable to be contacted by research staff (10)

▢ Their friends, family, or partner may want them to stop participating (11)

▢ Other (12) ________________________________________________

Skip To: End of Block If Selected Choices <= 3

Carry Forward Selected Choices from "What do you think are some of the challenges to getting people to come back for follow-up appointments? Select all that apply."

Select the top 3 challenges to getting people to come back for follow-up appointments.

| Top 3 Challenges: Getting people to come back for follow-up appointments |
| --- |
| ______ Not being able to get in touch with participants because their contact information changed (x1) |
| ______ Not being able to get in touch with participants because they gave false contact information when they started the study (x2) |
| ______ They may have trouble getting transportation for their appointments (x3) |
| ______ They may have trouble being able to show up at a specific appointment time (x4) |
| ______ They may have trouble getting to their appointment because of their work schedule (x5) |
| ______ They may have trouble finding childcare so that they can go to their appointment (x6) |
| ______ They may have concerns about confidentiality and privacy (x7) |
| ______ They may be afraid that the staff would judge them if they are still using drugs (x8) |
| ______ They may have stopped using drugs and no longer think the study is relevant to them (x9) |
| ______ They are in a drug treatment or recovery facility and are unable to be contacted by research staff (x10) |
| ______ Their friends, family, or partner may want them to stop participating (x11) |
| ______ Other (x12) |

Consider the top 3 challenges: ${RETCHAT/ChoiceGroup/SelectedChoices}. What could we do to overcome each of these?

________________________________________________________________

________________________________________________________________

________________________________________________________________

________________________________________________________________

________________________________________________________________

Q10.1 We are planning a study where we hope to increase follow-up appointment attendance by asking people in the study to help contact and remind others about their follow-up appointments. In this study, when people enroll and complete the first survey, they will get coupons to give to people they know who they think might qualify to participate. They will be paid when the people they recruit complete the survey, and they can refer up to 3 participants. When it comes time for the follow-up appointments six months later, we will ask the person who did the recruiting to help us contact the people they referred to the study and remind them about their follow-up appointments. For each of the three people who attend their follow-up appointment, the person who reminded them will get money (probably $10 per person).

What do you think some of the challenges of this process might be? Select all that apply.

▢ The person will have lost contact with the people they recruited (1)

▢ The contact information for the person they recruited may have changed and they may not be able to reach them (2)

▢ The person may have had a conflict with the person they recruited and not be talking with them now (3)

▢ They may not know the people they recruited well (4)

▢ Incentive of $10 per referred person may be too low (5)

▢ They will not want to waste minutes or data on their phone (6)

▢ The person who is supposed to help remind people or the people they are supposed to remind may be in recovery from substance use and trying not to talk with people who are actively using (7)

▢ The partner of the person they are calling may get jealous (8)

▢ Other (9) ________________________________________________

Skip To: End of Block If Selected Choices <= 3

Carry Forward Selected Choices from "What do you think some of the challenges of this process might be? Select all that apply."

Select the top 3 challenges for this recruitment process.

| Top 3 Challenges: Recruitment Process |
| --- |
| ______ The person will have lost contact with the people they recruited (x1) |
| ______ The contact information for the person they recruited may have changed and they may not be able to reach them (x2) |
| ______ The person may have had a conflict with the person they recruited and not be talking with them now (x3) |
| ______ They may not know the people they recruited well (x4) |
| ______ Incentive of $10 per referred person may be too low (x5) |
| ______ They will not want to waste minutes or data on their phone (x6) |
| ______ The person who is supposed to help remind people or the people they are supposed to remind may be in recovery from substance use and trying not to talk with people who are actively using (x7) |
| ______ The partner of the person they are calling may get jealous (x8) |
| ______ Other (x9) |

Consider the top 3 challenges: ${FEECHAT/ChoiceGroup/SelectedChoices}. What could we do to overcome each of these?

________________________________________________________________

________________________________________________________________

________________________________________________________________

________________________________________________________________

________________________________________________________________

Q11.1 In this study, we would offer people some training about how to encourage their friends to come back for their follow-up appointments. This training would involve a short video and then a staff person could answer follow-up questions.

What do you think this video should cover? Select all that apply.

▢ What to tell people about the follow-up appointments (e.g., how long they last, where they have to go for the appointment, how their information will be protected) (1)

▢ What the follow-up appointment involves (2)

▢ How they will get their money if people attend their follow-up appointments (3)

▢ How to talk to people about follow-up appointments without pressuring them (4)

▢ Ways to find them if their phone number or information has changed (5)

▢ What is in it for the person completing the follow up appointment (6)

▢ How to handle situations where the partner of the person they are calling gets jealous (7)

▢ Other (8) ________________________________________________

Skip To: VIDACT If Selected Choices <= 3

Carry Forward Selected Choices from "What do you think this video should cover? Select all that apply."

Select the top 3 topics for the training video.

| Top 3 Topics: Training Video |
| --- |
| ______ What to tell people about the follow-up appointments (e.g., how long they last, where they have to go for the appointment, how their information will be protected) (x1) |
| ______ What the follow-up appointment involves (x2) |
| ______ How they will get their money if people attend their follow-up appointments (x3) |
| ______ How to talk to people about follow-up appointments without pressuring them (x4) |
| ______ Ways to find them if their phone number or information has changed (x5) |
| ______ What is in it for the person completing the follow up appointment (x6) |
| ______ How to handle situations where the partner of the person they are calling gets jealous (x7) |
| ______ Other (x8) |

Should the video have actors or be animated?

o Actors (1)

o Animated (2)

Display This Question:If Should the video have actors or be animated? = Actors

VIDACTW What kind of actors should we have in the video?

________________________________________________________________

How long should the video be?

|  | Minutes |
| --- | --- |

|  | 0 | 1 | 2 | 3 | 4 | 5 | 6 | 7 | 8 | 9 | 10 |
| --- | --- | --- | --- | --- | --- | --- | --- | --- | --- | --- | --- |

| Length of Video () | 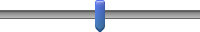 |
| --- | --- |

If we gave you a handout to take home with you after the video so that you could give it to a peer or remember what to tell a peer, what should it include? Select all that apply.

▢ What kind of questions will be asked during the follow-up appointments (1)

▢ Where follow-up appointments will be held (2)

▢ How long the follow up appointment will last (3)

▢ How long privacy and confidentiality are protected (4)

▢ Information about the staff person who will be doing their follow-up appointments (5)

▢ How much money people will receive for going to their follow-up appointment (6)

▢ How to talk to people about follow-up appointments without pressuring them (7)

▢ How to handle situations where the partner of the person they are calling gets jealous (8)

▢ Other (9) ________________________________________________

Carry Forward Selected Choices from "If we gave you a handout to take home with you after the video so that you could give it to a peer or remember what to tell a peer, what should it include? Select all that apply."

Select the top 3 items to include on a handout about the training video.

| Top 3 Items: Video Handout |
| --- |
| ______ What kind of questions will be asked during the follow-up appointments (x1) |
| ______ Where follow-up appointments will be held (x2) |
| ______ How long the follow up appointment will last (x3) |
| ______ How long privacy and confidentiality are protected (x4) |
| ______ Information about the staff person who will be doing their follow-up appointments (x5) |
| ______ How much money people will receive for going to their follow-up appointment (x6) |
| ______ How to talk to people about follow-up appointments without pressuring them (x7) |
| ______ How to handle situations where the partner of the person they are calling gets jealous (x8) |
| ______ Other (x9) |

OTHER What else do you think we should know as we begin this study?

________________________________________________________________

________________________________________________________________

________________________________________________________________

________________________________________________________________

________________________________________________________________

Q13.1 Thank you for the completing this survey!  Your information will help us to better understand issues impacting your community and to develop programs to improve health here.

STAFF [Staff to complete] Please enter comments about the interview here

________________________________________________________________

________________________________________________________________

________________________________________________________________

________________________________________________________________

________________________________________________________________
