# Supplementary material for: A cross-sectional survey of potential factors, motivations, and barriers influencing research participation and retention among people who use drugs in the rural USA
Source: Trials. 2021 Dec 20;22:948. doi: 10.1186/s13063-021-05919-w (PMC8690874; doi:10.1186/s13063-021-05919-w)
Supplement: Supplementary file 2 — Additional file 2: Potential factors, motivators, and barriers by study site. [file 13063_2021_5919_MOESM2_ESM.docx]

**Supplementary Material**

**Supplementary Figure 1.** Ranked influencing factors for participating in research among PWUD stratified by rural communities in Kentucky, Ohio, and Oregon, April 2019-July 2019


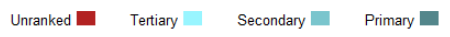

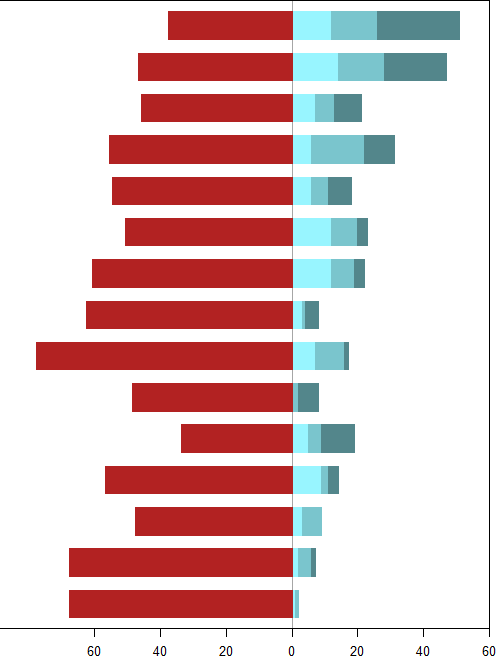


60 40 20 0 20 40

Count


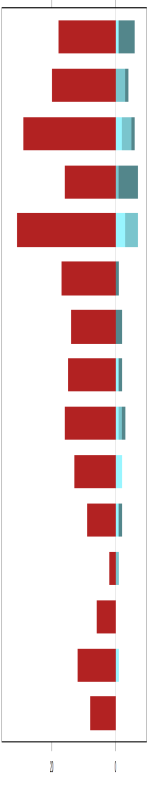


60 40 20 0 20 40

Count


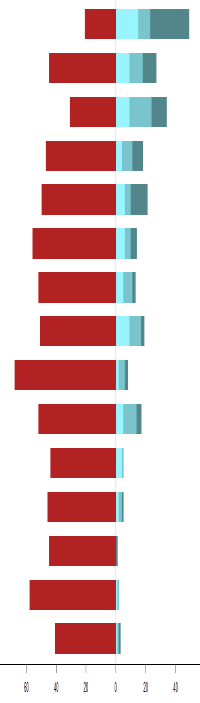


60 40 20 0 20 40

Count

|  | **Kentucky** | **Ohio** | **Oregon** |
| --- | --- | --- | --- |
| How much money they will receive |  |  |  |
| Whether their information will be kept confidential |  |  |  |
| How much time is required |  |  |  |
| Privacy of the research office |  |  |  |
| What the research study involves (e.g., survey, drug testing for research) |  |  |  |
| How far they have to travel to participate (i.e., nearby vs. out of town) |  |  |  |
| Why their information is being collected and what it will be used for |  |  |  |
| How much the project will benefit them overall |  |  |  |
| Whether the staff doing the research is friendly and trustworthy |  |  |  |
| How often they have to come in for visits |  |  |  |
| How their friends, family, or partner feels about them participating |  |  |  |
| Whether they have childcare so that they can attend their appointments |  |  |  |
| Whether their appointment times will interfere with their work schedule |  |  |  |
| Whether they can skip questions of parts of the study that make them uncomfortable |  |  |  |
| Whether the research institution or university is respected |  |  |  |

**Supplementary Figure 2.** Ranked motivators for joining a research study among PWUD stratified by rural communities in Kentucky, Ohio, and Oregon, April 2019-July 2019


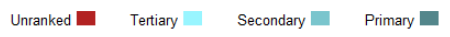

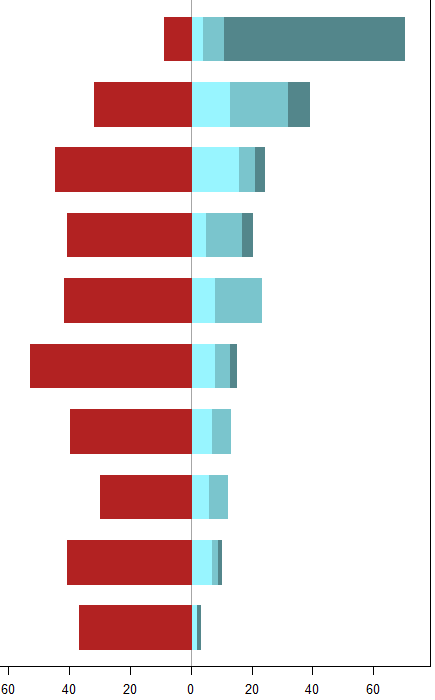


60 40 20 0 20 40 60

Count


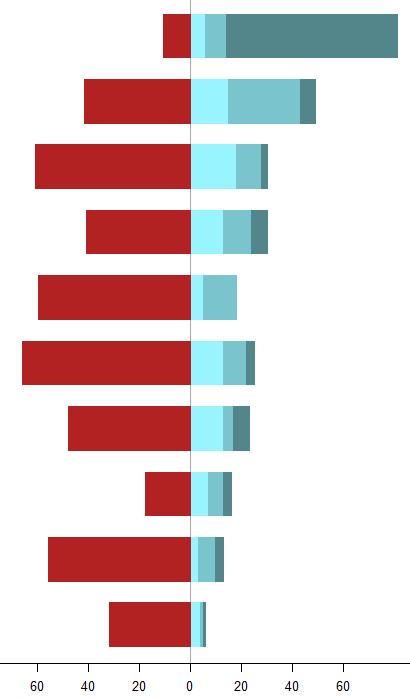


60 40 20 0 20 40 60

Count


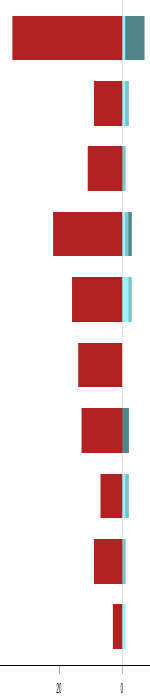


40 20 0 20 40

Count

|  | **Kentucky** | **Ohio** | **Oregon** |
| --- | --- | --- | --- |
| Financial incentive (i.e., money or gift card given for participation |  |  |  |
| They would want to get free testing (for example, rapid tests for HIV & HCV) if it was offered as part of the study |  |  |  |
| They would want to be linked with resources and/or follow-up testing if it was offered as part of the study |  |  |  |
| They believe in the mission of the research and want to contribute |  |  |  |
| Their friends, family, or partner participates |  |  |  |
| They would want to try a new treatment if it was offered as part of the study |  |  |  |
| They want to tell their story |  |  |  |
| Their friends, family, or partner pressures them to participate so that they can share the financial incentive |  |  |  |
| They want to learn about the topic |  |  |  |
| They know someone on the research team and want to help them out |  |  |  |

**Supplementary Figure 3.** Ranked barriers to returning to follow-up research appointments among PWUD stratified by rural communities in Kentucky, Ohio, and Oregon, April 2019-July 2019


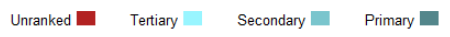

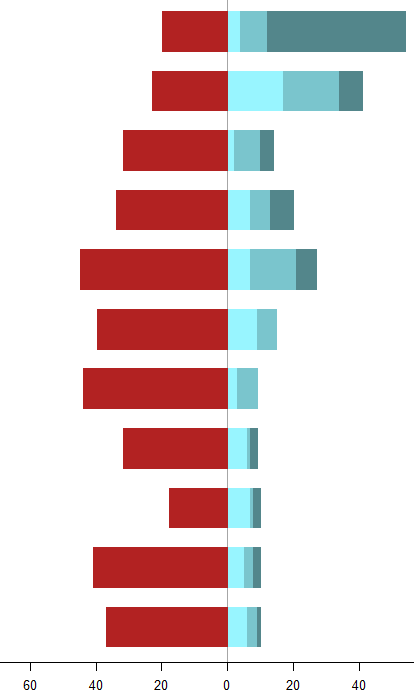


60 40 20 0 20 40 60

Count


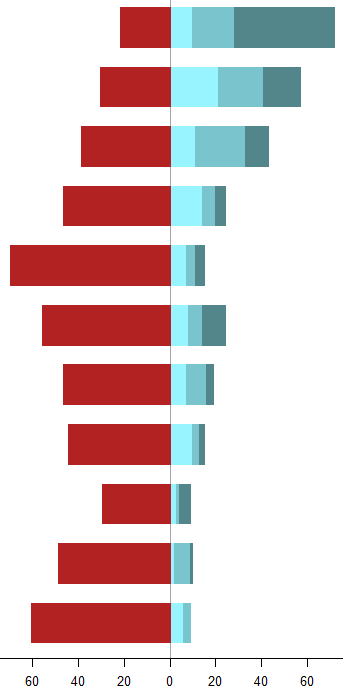


60 40 20 0 20 40 60

Count


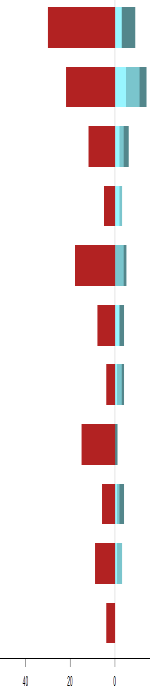


60 40 20 0 20

Count

|  | **Kentucky** | **Ohio** | **Oregon** |
| --- | --- | --- | --- |
| Not being able to get in touch with participants because their contact information changed |  |  |  |
| They may have trouble getting transportation for their appointments |  |  |  |
| Not being able to get in touch with participants because they gave false contact information when they started the study |  |  |  |
| They may have concerns about confidentiality and privacy |  |  |  |
| They may have trouble being able to show up at a specific appointment time |  |  |  |
| They are in a drug treatment or recovery facility and are unable to be contacted by research staff |  |  |  |
| They may have trouble finding childcare so that they can go to their appointment |  |  |  |
| They may be afraid that the staff would judge them if they are still using drugs |  |  |  |
| Their friends, family, or partner may want them to stop participating |  |  |  |
| They may have trouble getting to their appointment because of their work schedule |  |  |  |
| They may have stopped using drugs and no longer think the study is relevant to them |  |  |  |
